# The auditory phenotype of behavioural variant and right temporal variant frontotemporal dementia

**DOI:** 10.1002/alz.091457

**Published:** 2025-01-03

**Authors:** Jeremy Johnson, Benjamin Aaron Levett, Mai‐Carmen Requena‐Komuro, Elia Benhamou, Jessica Jiang, Doris‐Eva Bamiou, Chris JD Hardy, Jason D Warren

**Affiliations:** ^1^ King’s College London, London United Kingdom; ^2^ Dementia Research Centre, UCL Queen Square Institute of Neurology, University College London, London, London United Kingdom; ^3^ Dementia Research Centre, Institute of Neurology, University College London, London United Kingdom; ^4^ Dementia Research Centre, Department of Neurodegenerative Disease, UCL Queen Square Institute of Neurology, University College London, London United Kingdom; ^5^ University College London, London United Kingdom; ^6^ Dementia Research Centre, UCL Queen Square Institute of Neurology, University College London, London United Kingdom

## Abstract

**Background:**

Patients with behavioural variant frontotemporal dementia (bvFTD) and right temporal variant frontotemporal dementia (rtvFTD) commonly exhibit abnormal hedonic and other behavioural responses to sounds, however hearing dysfunction in this disorder is poorly characterised. Here we addressed this issue using the Queen Square Tests of Auditory Cognition (QSTAC) – a neuropsychological battery for the systematic assessment of central auditory functions (including pitch pattern perception, environmental sound recognition, sound localisation and emotion processing) in cognitively impaired people.

**Method:**

The QSTAC was administered to 12 patients with bvFTD, 7 patients with rtvFTD and 24 patients with comparator dementia syndromes (primary progressive aphasia and typical Alzheimer’s disease) and 15 healthy age‐matched individuals. Participants also underwent pure tone audiometry to assess peripheral hearing function and a comprehensive general neuropsychological assessment.

**Result:**

After accounting for nonverbal executive and peripheral hearing performance, patients with bvFTD and rtvFTD showed deficits of environmental sound and auditory emotion recognition and sound localisation, compared both with healthy controls and patients with Alzheimer’s disease. Patients with bvFTD showed a deficit in dichotic listening compared with the rtvFTD group.

**Conclusion:**

bvFTD and rtvFTD have distinct phenotype of auditory cognitive dysfunction which likely contributes to the hearing alterations that many patients with this diagnosis experience in daily life. Our findings call attention to an under‐recognised issue in frontotemporal dementia that warrants further clinical interpretation and the development of management strategies tailored to real‐world acoustic environments.

